# Acute kidney injury of all severity is associated with extended hospitalization after critical illness

**DOI:** 10.1186/cc13558

**Published:** 2014-03-17

**Authors:** JR Prowle, I Kolic, J Purdell-Lewis, CJ Kirwan

**Affiliations:** 1 Barts Health NHS Trust, London, UK

## Introduction

Acute kidney injury (AKI) complicates over 50% of ICU admissions and is associated with significantly increased mortality, length of stay, and costs across a broad spectrum of conditions [1].

## Methods

We performed a single-centre, retrospective analysis of AKI diagnosis in patients with ICU admissions of 5 days or more who survived to hospital discharge between 2009 and 2011. We examined the relationship between hospital length of stay, AKI diagnosis, demographics and clinical characteristics in a multivariable Cox-hazard analysis.

## Results

**Figure 1:**
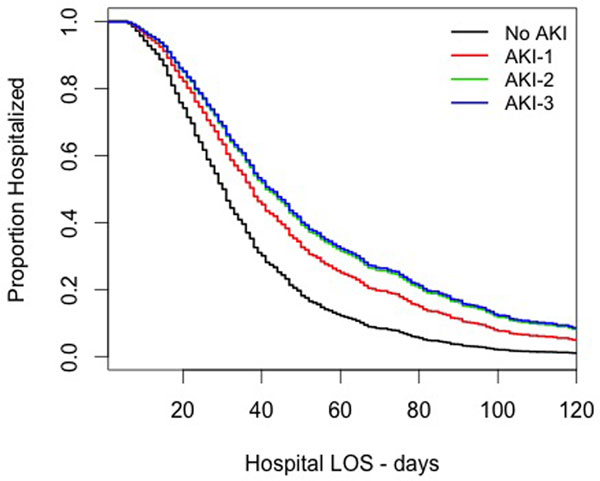
**Cox-hazard prediction of AKI class on hospitalization (ICU LOS fixed at median)**.

We identified 700 cases, with a 66% incidence of AKI. The AKI was associated with older age, greater initial illness severity and longer ICU and hospital length of stay in univariate analysis (Table 1). In Cox-hazard analysis, only AKI category and ICU length of stay were significantly associated with lower probability of discharge over time (Figure 1). AKI-1 was associated with a hazard ratio for hospital discharge of 0.66 (0.55 to 0.79), AKI-2 with 0.55 (0.42 to 0.71) and AKI-3 with 0.54 (0.44 to 0.66).

**Table 1:** Patient characteristics by AKI

	No AKI	AKI	*P *value
Age	46 (32 to 60)	51 (37 to 64)	<0.001
SAPS-2	35 (27 to 42)	41 (32 to 49)	<0.001
ICU LOS	8 (6 to 12)	12 (7 to 18)	<0.001
Hospital LOS	27 (17 to 42)	41 (28 to 72)	<0.001

Data presented as median (IQR).

## Conclusion

AKI was a significant predictor of remaining in hospital at all levels of AKI severity even after allowing for longer ICU stay. Even mild AKI is associated with extended recovery from critical illness and healthcare costs even after ICU discharge.
